# Decision Making and Impulsivity in Young Adult Cannabis Users

**DOI:** 10.3389/fpsyg.2021.679904

**Published:** 2021-07-01

**Authors:** Brian F. O'Donnell, Patrick D. Skosnik, William P. Hetrick, Daniel J. Fridberg

**Affiliations:** ^1^Department of Psychological and Brain Sciences and Program in Neuroscience, Indiana University, Bloomington, IN, United States; ^2^Department of Psychiatry, Yale University School of Medicine, New Haven, CT, United States; ^3^Department of Psychiatry and Behavioral Neuroscience, The University of Chicago, Chicago, IL, United States

**Keywords:** cannabis, decision making, impulsivity, delay discounting, Iowa Gambling Task, probabilistic reversal learning

## Abstract

**Aims:** Chronic cannabis users show impairments on laboratory measures of decision making which reflect risk factors for initiation and continued use of cannabis. However, the differential sensitivity of these tasks to cannabis use has not been established. Moreover, studies to date have often lacked assessment of psychiatric histories and use of other illicit substances, both of which may influence decision making outcomes. The current study aimed to address these limitations by (1) including multiple types of decision making tasks, (2) implementing the Probabilistic Reversal Learning Task, a measure of decision making under uncertainty, for the first time in cannabis users, (3) including young adult cannabis users with no other psychiatric disorders, and (4) conducting urinalysis to exclude users of other illicit drugs.

**Methods:** Thirty-three current cannabis users without comorbid psychiatric disorders and 35 cannabis non-users completed behavioral measures of decision-making (Iowa Gambling Task), reward discounting (Delay Discounting Task), choice-outcome learning (the Probabilistic Reversal Learning Task) and a questionnaire assessment of impulsivity (Barratt Impulsiveness Scale).

**Results:** Relative to non-users, cannabis users demonstrated greater preference for immediate vs. delayed rewards on the Delay Discounting Task, made fewer advantageous decisions on the Iowa Gambling Task, and endorsed greater impulsivity on the Barratt Impulsiveness Scale scales. Cannabis users and non-users showed comparable performance on the Probabilistic Reversal Learning Task. Frequency of past-month cannabis use and total years of use did not predict decision making or impulsivity.

**Conclusions:** Young adult cannabis users demonstrated higher discounting rates and impairments in learning cost-benefit contingencies, while reversal learning was unaffected. Self-reported impulsivity was elevated as well. None of these measures correlated with current or lifetime estimates of cannabis use, arguing against a dose-relationship. Interventions that target improvement in affected components of decision making may be helpful in reducing cannabis use and relapse.

## Introduction

Approximately 43.5 million Americans used cannabis in 2018, making it third most popular substance used in the United States behind alcohol and tobacco, with 1.6% of users meeting past-year criteria for cannabis use disorder (SAMHSA, 2019). Repeated exposure to delta 9-tetrahydrocannabinol, the primary psychoactive constituent of cannabis, alters activity at endogenous cannabinoid type 1 receptors. Cannabinoid type 1 receptors are densely distributed in brain structures involved in decision making and reward learning, including the hippocampus, amygdala, cingulate cortex, prefrontal cortex, cerebellum, dorsal striatum and ventral tegmental area (Glass et al., 1997; Eggan and Lewis, 2007; Parsons and Hurd, 2015; Curran et al., 2016). Decision making is a multi-stage process whereby individuals evaluate potential options, form preferences, select and execute actions, and react to the outcome of actions (Ernst and Paulus, 2005). Chronic cannabis users show deficits on a variety of decision making tasks (Bechara, 2005; Crean et al., 2011; Fatima et al., 2019). Nevertheless, the differential sensitivity of various decision making tasks in the context of cannabis use has not been established.

To address these issues, the present study compared cannabis users and cannabis-non-users on three behavioral tasks intended to capture different aspects of decision making: the Iowa Gambling Task, IGT (Bechara et al., 1994), Delay Discounting Task (Rachlin et al., 1991; Amlung et al., 2017) and the Probabilistic Reversal Learning Task (PRLT (Izquierdo et al., 2017). The IGT is a decision-making paradigm wherein participants must integrate outcomes (gains or losses of money) from a series of risky choices over time to guide future decision-making. Prior studies have shown that chronic cannabis users perform worse on the IGT relative to non-users (Whitlow et al., 2004; Lamers et al., 2006; Fridberg et al., 2010; Casey and Cservenka, 2020), though not all studies have found this impairment (Dougherty et al., 2013; Verdejo-Garcia et al., 2013). For the Delay Discounting Task, participants make repeated choices between small rewards delivered immediately or larger rewards delivered after some delay, with the rate at which the larger rewards are discounted as a function of time to their receipt providing a measure of impulsive decision making (Mischel et al., 1989; Bickel et al., 2019). Mejia-Cruz et al. (2016) found that cannabis dependent users showed higher discounting rates for delayed monetary losses than non-users, while others have found no difference between groups (Strickland et al., 2017; Jarmolowicz et al., 2020). These differences among studies may be influenced by variations in cannabis use among samples. Greater frequency of cannabis use (Sofis et al., 2020), greater substance use symptom severity (Stea et al., 2011; Strickland et al., 2017) and earlier age of onset of use (Kollins, 2003; Heinz et al., 2013) have been associated with increased discounting rates among cannabis users. The PRLT is an operant learning paradigm in which participants make repeated choices between two stimuli on each trial, with immediate feedback (e.g., a gain or loss of points) determined probabilistically (e.g., the “correct” and “incorrect” stimuli may result in a gain of points only 80 or 20% of the time, respectively) (Cools et al., 2002). The contingency reverses multiple times during the task (i.e., the usually correct stimulus becomes usually incorrect, and vice-versa), requiring participants to integrate feedback regarding his or her choices over multiple trials and adapt responses appropriately to select the correct stimulus. While discrimination reversal learning paradigms have revealed behavior deficits in animal models of addiction (Izquierdo and Jentsch, 2012), including acute delta 9-tetrahydrocannabinol administration (Egerton et al., 2005; Wright et al., 2013), to our knowledge no prior published studies have examined the performance of human cannabis users on the PRLT. Rimonabant, a CB1 receptor antagonist/inverse agonist, has been reported to improve PRLT performance in persons with schizophrenia (Boggs et al., 2012), suggesting that modulation of CB1 receptors in humans may impact PRLT performance.

Impulsivity is a broad construct comprising multiple dimensions, including inability to delay gratification, sensation seeking, risk-taking and insensitivity to long term consequences. In clinical populations, impulsivity is typically assessed using a self-report questionnaire such as the Barratt Impulsiveness Scale (BIS; Patton et al., 1995). Self-report impulsivity is usually increased in persons with substance use disorders (Stanford et al., 2009). Delay Discounting and the IGT are often conceptualized as tasks which test cognitive mechanisms associated with impulsivity, although correlations between laboratory and self-report tasks are small in magnitude (Duckworth and Kern, 2011; Barnhart and Buelow, 2017; Ellingson et al., 2018; Creswell et al., 2019), suggesting that self-report impulsivity taps facets of impulsivity that are dissociable from laboratory task performance. BIS scores have been reported to be elevated in both current cannabis users (Dougherty et al., 2013) and in abstinent former users (Delibas et al., 2018). Among cannabis users, self-reported impulsivity predicted more frequent cannabis use (Vangsness et al., 2005). The BIS was used in the current study to assess trait impulsivity.

Of note, while decision making impairments have been reported previously in cannabis use disorder, interpretation of these findings is often limited by potential confounds in sample characteristics. Population samples indicate that persons with cannabis dependence have very high lifetime rates of psychiatric comorbidity, approaching 90% (Agosti et al., 2002), which may contribute to decision making deficits. However, studies of decision making in cannabis users often have not assessed or excluded subjects for lifetime history of psychiatric disorders, as in previous studies of delay discounting in that population (Stea et al., 2011; Boggs et al., 2012; Heinz et al., 2013; Peters et al., 2013; Lee et al., 2015; Aston et al., 2016; Strickland et al., 2017; Jarmolowicz et al., 2020; Sofis et al., 2020). A second issue in studies of discounting rates is lack of urinalysis screening for illicit drug use in cannabis users to rule out other substance use and, in control participants, to confirm self-reported abstinence from cannabis or illicit substances (Johnson et al., 2010; Stea et al., 2011; Heinz et al., 2013; Peters et al., 2013; Strickland et al., 2017; Jarmolowicz et al., 2020). In the present study, assessment of current and past psychopathology as well drug screening were included in the inclusion criteria to minimize the potential influences of those variables on study outcomes.

For the present study, we hypothesized that cannabis users without a history of comorbid psychiatric disorders would exhibit more disadvantageous decision making on the IGT, steeper discounting of delayed rewards on the PRLT, and greater difficulty acquiring and reversing contingencies on the PRLT. For these tests, a significance threshold of *p* < 0.05 was used. We also hypothesized that cannabis users would score higher than non-users on the total score of the BIS self-report measure and the three second order BIS factor scores. Exploratory analyses were used to evaluate whether measures of current and past cannabis use contributed to increased impulsivity or decision making impairments in cannabis users, using a threshold of *p* < 0.01.

## Methods

### Participants

Thirty-three current cannabis users (7 females) and 35 cannabis-non-users (i.e., no lifetime cannabis use; 14 females) completed the study. Participants were recruited from the local university community and were paid $10 per hour for their participation. The study protocol was approved by the Indiana University Human Subjects Committee and written informed consent was obtained from all participants.

Inclusion criteria were as follows: (1) For the cannabis-using group: current cannabis consumption at the rate of at least once per week (mean ± SD = 5.6 ± 1.7 times/week), no other illicit substance use during the past 3 months, and no DSM-IV diagnosis of Axis I or II disorders except cannabis abuse or dependence; (2) For the cannabis non-user group: no history of any illicit substance use and no history of psychiatric illness (Axis I or II); (3) For all participants: ages 18–35, completed a high school education, no history of cardiovascular disease, hearing problems, neurological disease, learning disability, or head injury resulting in loss of consciousness, and self-reported alcohol consumption <3 standard drinks per day for males and 2 standard drinks per day for females. Subjects were not excluded for tobacco use. Cannabis users were asked to abstain from cannabis use for 24 h prior to participating in the study.

### Clinical Interviews and Drug Use Assessment

The Structured Clinical Interview for DSM-IV Axis I and II Disorders (SCID-I and SCID-II; First et al., 1997, 2002) were used to rule out previous psychiatric conditions and assess drug use histories. In addition, a locally developed drug-use questionnaire was used to ascertain current and past cannabis consumption patterns including age of first use of cannabis, total years of use and frequency of use over the past week and month. The drug-use questionnaire was based on a time-line follow back approach and was used to ascertain cannabis use patterns as described previously (Skosnik et al., 2012, 2014). Briefly, levels of cannabis consumption (estimated number of joints) were determined for lifetime, the past six 6, 3 months, 1 month, and then for the week before the test session. Participants were instructed to consider each day of the week and indicate, for an average week, how much they consumed per drug-use occasion for each length of time assessed. Questionnaire items are available in the Supplementary Material.

Self-reported recent drug use was corroborated using urine drug screens (Q10-1, Proxam). Participants included in the non-user group tested negative for all illicit drugs, whereas those included in the cannabis-using group tested positive for cannabis only. Subjects were administered the American National Adult Reading Test (ANART; Strauss et al., 2006) to estimate verbal IQ (Gladsjo et al., 1999). The Barratt Impulsiveness Scale (BIS) was used to assess trait impulsiveness and three second-order factor scores (Patton et al., 1995). The total score of the BIS has good internal consistency (Cronbach's alpha = 0.83) with lower alpha values for the factor scores of the Attention (0.74), Motor (0.59) and Non-Planning (0.72) subscales (Stanford et al., 2009). Demographic, verbal IQ and cannabis use information for the control and cannabis-using groups are presented in Table 1. Twenty-four members of the cannabis-using group met DSM-IV criteria for cannabis abuse or dependence.

**Table 1:** Demographic, IQ, BIS, and cannabis use information for the control and cannabis-using groups.

		**Control (*n* = 35)**	**Cannabis-using (*n* = 33)**
		**Mean (SD)**	**Mean (SD)**
Sex	Female (*n*)	14	7
	Male (*n*)	21	26
Age		21.0 (3.0)	20.1 (2.7)
Education		**14.0 (1.4)**	13.1 (1.3)*
Est. verbal IQ (ANART)		107.1 (4.7)	106.8 (4.3)
Freq. of cannabis use (exposures past month)		–	49.0 (38.2)
Age of first cannabis use (years)		–	16.0 (2.2)
Cannabis use in years		–	4.2 (3.6)
**BIS**			
Attentional		**14.0 (3.3)**	15.9 (3.0)*
Motor		**20.7 (3.4)**	21.6 (3.5)*
Non-planning		**22.1 (5.0)**	24.8 (4.4)*
Total score		**56.7 (9.9)**	62.3 (9.2)*

*BIS, Barrett Impulsiveness Scale. Values in bold with an asterisk indicate significant group difference at p < 0.05*.

### Behavioral Task Methods

Detailed methods for the three decision making tasks are provided in Supplementary Materials.

### Iowa Gambling Task

A computerized version of the IGT (Bechara et al., 1994) required participants to choose among four decks of cards which were displayed face down on the screen. Each selection was associated with either a win of $100 (Decks A or B) or $50 (Decks C or D). Furthermore, 50% of selections from Deck A and Deck C were paired with small, frequent losses, whereas 10% of selections from Deck B and Deck D were paired with larger, less frequent losses. Decks C and D were advantageous decks that resulted in a net gain of $250 across 10 selections, whereas selecting from Decks A and B (the disadvantageous decks) resulted in a net loss of $250 across 10 selections. Feedback regarding the amount won or lost was displayed after each selection, allowing for learning over the course of the task. Participants completed 150 selections during the IGT, which was divided into 3 blocks of 50 trials for data analysis. One hundred fifty trials were used, as in several previous studies (Steingroever et al., 2015), in order to more fully characterize the learning curve in both groups. For each participant, a “net score” (defined as the number of selections from advantageous decks minus the number of selections from disadvantageous decks) was calculated as an index of IGT performance for each block of 50 trials. IGT data from 11 control and 3 cannabis-using members were excluded from analysis because those individuals completed a pilot version of the task which used a different payout schedule than that used in the final study.

### Delay Discounting Task

Participants completed a computerized version of the Delay Discounting Task. On each trial, participants were presented with a choice between an amount of money available immediately or a larger amount of money available after some delay. The amount of the delayed reward was $800 for each condition. The amount of immediate reward and delayed period were varied across conditions using an adjusting amount procedure (Green et al., 2005) to converge on an estimate of an immediately-available amount of money that the subject considered equivalent to the delayed amount. The task consisted of six delay conditions presented in random order, and participants made six choices per condition. The data from each participant was fit to the following hyperbolic delay discounting function (Mazur, 1987; Green and Myerson, 2004):

$$\begin{array}{l} V=A/\left(1+kD\right), \end{array}$$

where *A* is the amount of the delayed reward (in the present study, $800), *D* is the delay until its receipt, *V* is subjective equivalent value of an immediate reward, and *k* is a free parameter corresponding to the individual's discounting rate. Higher values of *k* correspond to steeper discounting of delayed rewards and a greater preference for immediate rewards. Discounting rates (*k*) were transformed using the natural logarithm prior to analysis (Kirby et al., 1999).

### Probabilistic Reversal Learning Task

For the PRLT, participants selected one of two stimuli (different colored squares) on each trial to determine, based upon feedback, which stimulus was usually correct. Each stimulus was associated with a gain (if correct) or loss (if incorrect) of 100 points. Participants were instructed to choose the stimulus within each pair that was usually correct and to update their response accordingly should the usually-correct stimulus become usually-incorrect (and vice versa), and that their goal was to win as many points as possible during the task. The feedback conditions were 100:0, 80:20, or 70:30, indicating the ratio of positive:negative (i.e., accurate:misleading) feedback provided upon selection of the correct stimulus from that pair. Each of the three stimulus pairs (feedback conditions) were presented for 80 trials. The criterion for demonstrating successful learning of each contingency condition was eight consecutive correct responses to that pair. After that criterion was met, the probability was 0.25 that the pair would reverse on the subsequent presentation. Following reversal, eight consecutive correct responses to the pair were required before it reversed again. Outcome measures for the PRLT were derived separately for each feedback condition (100:0, 80:20, and 70:30) and included: the number of trials required to learn the contingency (trials to acquisition), the total number of errors and the total number of reversals completed successfully.

### Hypotheses and Statistical Analysis

Relative to non-using group, we hypothesized that cannabis users would show: (1) lower net scores across trial blocks on the IGT; (2) more rapid temporal discounting of monetary rewards (i.e., higher values of *k*) on the Delay Discounting Task; (3) fewer successful reversals and more errors on the PRLT; and (4) elevated impulsivity on the BIS total and second-order factor (subscale) scores. Analysis of covariance (ANCOVA) with the covariates sex (female = 0; male = 1) and years of education were used to test for group differences and group interactions for analysis of task variables and BIS scores. The Huynh-Feldt correction was applied when the Mauchly's Sphericity Test was significant (*p* < 0.05) for repeated measures ANCOVA. Pearson correlation coefficients were used in exploratory analyses to evaluate associations between cannabis use, decision making performance and impulsivity measures.

## Results

### Participants

Demographic, IQ, BIS and cannabis use information for the participants are provided in Table 1. Groups did not significantly differ in age [*F*_(1, 66)_ = 1.37, *p* = 0.25], estimated IQ [*F*_(1, 65)_ = 0.07, *p* = 0.80] or in sex distributions [χ^2^(1) = 2.81, *p* = 0.09]. Cannabis users had about 1 year less educational attainment than non-users [*F*_(1, 66)_ = 4.14, *p* < 0.01]. The mean age of initiation of cannabis use was 16.0 years, and the mean frequency of use in the past month was 49 exposures.

### Iowa Gambling Task

The mean net scores for the control and cannabis-using groups on each block of the IGT are shown in Table 2. A repeated measures ANCOVA with the factors Group (2) X Block (3) and the covariates of education and sex revealed a significant Group X Block interaction, *F*_(1.87, 104)_ = 3.33, *p* = 0.02, with a non-significant main effect of Group, *F*_(1, 50)_ = 0.99, *p* = 0.32. *Post-hoc* ANOVAs indicated that the cannabis group made significantly fewer advantageous decisions than the control group during Block 2 of the task, *F*_(1, 52)_ = 7.42, *p* = 0.01. A main effect of block for the IGT indicated that participants learned to choose more from the advantageous decks as the task progressed, *F*_(1.87, 104)_ = 18.83, *p* < 0.001.

**Table 2:** Mean (SD) net scores on the Iowa Gambling Task for the control and cannabis-using groups.

	**Control (*n* = 24)**	**Cannabis-using (*n* = 30)**
Block 1	−3.17 (18.14)	−3.47 (13.51)
Block 2	**14.83 (24.71)**	−0.33 (16.03)*
Block 3	17.50 (24.05)	11.82 (23.24)

*Each block included data from 50 trials. Values in bold with an asterisk indicate significant group difference at p < 0.05*.

### Delay Discounting Task

Log-transformed discounting rates were compared between the groups using an ANCOVA with the covariates of education and sex. One participant from the control group and one participant from the cannabis using group were identified as outliers based upon their discounting rates (i.e., individual *k* values >3 *SD*s above or below their respective group means) and were excluded from the analyses of Delay Discounting Task data. The analysis revealed that the cannabis group exhibited greater discounting of delayed rewards relative to the control group, control mean (*SD*) ln *k* = −5.05 (1.37); cannabis group mean = −4.31 (1.40); *F*_(1, 63)_ = 4.41, *p* = 0.04.

### Probabilistic Reversal Learning Task

Six control subjects and five cannabis-using subjects failed to meet the learning criterion (eight consecutive correct responses) on one or more contingency conditions and were excluded from further PRLT analyses. Group differences in trials to acquisition, total number of errors and number of reversals were assessed across task version and contingency conditions using Group (2) X Contingency Condition (3) repeated measure ANCOVAs with the covariates of education and sex (see Table 3). There was a significant Group X Contingency interaction for total errors on the PRLT [*F*_(1.72, 94.66)_ = 4.14, *p* = 0.02], indicating that the control group committed more errors than the cannabis using group for the 80:20 contingency condition [*F*_(1, 55)_ = 6.60, *p* = 0.01]. A main effect of Contingency Condition was significant for trials to acquisition [*F*_(1.36, 74.72)_ = 13.47, *p* < 0.001], total errors [*F*_(1.72, 94.66)_ = 224.38, *p* < 0.001] and number of reversals [*F*_(2.00, 110)_ = 144.30, *p* < 0.001], showing that performance declined with increased task difficulty. The main effect of group was not significant for trials to acquisition [*F*_(1, 53)_ = 3.04, *p* = 0.09], total errors [*F*_(1, 53)_ = 0.18, *p* = 0.68], or number of reversals [*F*_(1, 53)_ = 0.11, *p* = 0.75]. The Group X Contingency interaction was not significant for trials to acquisition [*F*_(1.36, 74.72)_ = 0.97, *p* = 0.35] or number of reversals [*F*_(2.00, 110.0)_ = 0.41, *p* = 0.53].

**Table 3:** Mean (SD) outcomes on the probabilistic reversal learning task (PRLT) for the control and cannabis-using groups, by task contingency condition.

**Outcome**	**Group**	**Contingency condition**		
		**100:0**	**80:20**	**70:30**
Trials to acquisition	Control	9.3 (2.8)	11.8 (5.1)	16.2 (14.2)
	Cannabis-using	10.1 (3.8)	16.5 (8.8)	21.3 (14.8)
Reversals	Control	5.6 (0.9)	3.8 (1.1)	2.8 (1.2)
	Cannabis-using	5.7 (1.0)	3.8 (1.4)	2.6 (1.1)
Total errors	Control	10.4 (2.7)	22.2 (4.3)	28.3 (7.0)
	Cannabis-using	10.8 (2.2)	19.1 (4.9)*	30.1 (7.6)

*Values in bold with an asterisk indicates significant group difference at p < 0.05*.

### Barratt Impulsiveness Scale

BIS scores are displayed in Table 1. The cannabis using group scored higher than the control group on the BIS total score [*F*_(1, 64)_ = 5.23, *p* = 0.03]. A repeated measures ANCOVA with the factors Group (2) and Subscale (3: Attentional, Non-Planning, Motor) and the covariates of education and sex revealed a main effect of Group [*F*_(1, 64)_ = 5.23, *p* = 0.03], indicating an elevation of impulsivity scores across subjects. The interaction of Group X Subscale was not significant [*F*_(2, 132)_ = 1.52, *p* = 0.22].

### Relationship of Cannabis Use to Decision Making and Impulsivity

Correlation coefficients were used to evaluate potential associations among cannabis use (frequency of use in the past month, total years of use), measures of decision making that were impaired in cannabis users (IGT net score, Delay Discounting ln *k*), and impulsivity (BIS Total Score; see Supplementary Materials). None of the correlation coefficients between the cannabis use measures and the behavioral measures were significant at *p* < 0.01.

## Discussion

The goal of the present study was to compare healthy current cannabis users to non-cannabis using control subjects on behavioral and personality measures relevant to decision making. Cannabis users had no history of comorbid psychiatric disorders and all subjects were screened using urinalysis to confirm self-report of cannabis and other illicit drug use. Relative to non-users, cannabis users made poorer decisions on the Iowa Gambling Task, showed greater discounting of hypothetical delayed rewards on the Delay Discounting Task and scored higher on the Barratt Impulsiveness Scale, a questionnaire measure of trait impulsivity. In contrast, cannabis users were not impaired in performance on the PRLT. Among cannabis users, there were no significant correlations between frequency of past-month cannabis use and total years of use and outcomes on the decision making tasks or BIS. Collectively, the present data show that cannabis users without psychiatric comorbidities or diminished verbal IQ may still exhibit heightened impulsivity and worse decision making on two well-validated laboratory decision making tasks, relative to non-using control group. These findings also highlight the potential for targeting decision making in interventions for cannabis use disorders (Verdejo-Garcia et al., 2007; Vassileva and Conrod, 2019).

Cannabis users made worse decisions over time than did non-users on the IGT, in line with previous studies (Whitlow et al., 2004; Lamers et al., 2006; Fridberg et al., 2010; Casey and Cservenka, 2020). Importantly, learning was not entirely absent among cannabis users in the present study. In the current study, cannabis users demonstrated learning on the IGT and favored advantageous decks by the final block of the task. This suggests that cannabis users may take longer than non-users to learn the relations between choices and associated long-term outcomes. Cognitive modeling analysis of cannabis users' choices on the IGT suggests that users rely upon recent outcomes to a greater extent than controls when choosing cards on the IGT (Fridberg et al., 2010). This could explain the “learning lag” exhibited by cannabis users on the IGT, and suggests that cannabis users may be more prone to forgetting past outcomes of decisions than non-users (Fridberg et al., 2010) which could contribute to poorer decision making over time.

Cannabis users also showed steeper discounting of delayed hypothetical monetary rewards on the Delay Discounting Task relative to controls, suggesting that decision-making in cannabis users may be influenced by their greater preference for immediate rewards. Cannabis users' greater discounting rates on the Delay Discounting Task may therefore indicate a greater tendency toward impulsive decision making, relative to non-using individuals. Our findings replicate Mejia-Cruz et al. (2016), but null findings have also been reported (Strickland et al., 2017; Jarmolowicz et al., 2020). Of note, Johnson et al. (2010) reported a trend for increased discounting rates in current, but not in abstinent former cannabis users. Longitudinal studies that encompass periods of use and abstinence in cannabis users could clarify these relationships.

We did not find support for reversal learning deficits among cannabis users on the PRLT, suggesting that this task is less sensitive to cannabis use than the IGT or delay discounting. The PRLT measures the ability to learn stimulus-outcome contingencies over multiple decisions and adjust one's behavior following changes in those relations, which is hypothesized to be an important component of effective decision making (Rahman et al., 2001; Clark et al., 2004; Fellows and Farah, 2005). While the IGT also requires decision-makers to integrate feedback over multiple decisions to guide their choices, there are plausible explanations for why cannabis users in the present study showed deficits on the IGT but not the PRLT. For instance, cannabis users may be able to recruit additional cognitive resources which aid their decision-making process during simpler learning challenges like the PRLT. Indeed, performance on a complex decision-making task such as the IGT could be affected by multiple additional factors, including impulsivity and sensitivity to rewards vs. punishments (Busemeyer and Stout, 2002; Yechiam et al., 2005). This may explain why users appeared to display a “learning lag” on the IGT relative to non-users, but did not differ significantly from controls with regard to the number of trials required to learn each contingency pair on the PRLT. Another possible explanation for the differences in performance between cannabis users and controls across the IGT and PRLT is that reversal learning deficits may emerge only after many years of chronic cannabis use. Importantly, the cannabis users in the present study were not intoxicated during testing, in contrast to studies which have demonstrated that acute administration of delta 9-tetrahydrocannabinol produces deficits in reversal learning paradigms in both rats (Egerton et al., 2005) and rhesus macaques (Wright et al., 2013). It is possible that PRLT would be affected in humans during cannabis intoxication but unaffected by chronic use per se.

Cannabis users endorsed greater trait impulsivity than controls in the present study, as evidenced by higher scores on the BIS overall and increased levels of the second order factors. Dougherty et al. (2013) reported increased BIS scores in current cannabis users as well, suggesting that these elevations are a robust characteristic in samples of cannabis users. Delibas et al. (2018) found that the total BIS score and Non-Planning Impulsivity were elevated in former cannabis users, suggesting that these elevations were not due to recent use. Impulsivity was not correlated with measures of cannabis use, convergent with other evidence that impulsivity is primarily a risk factor rather than a consequence of substance use (Verdejo-García et al., 2008).

None of the measures affected in cannabis users were correlated with recent use or with duration of use, arguing against a dose-response relationship. This finding replicates previous studies which did not find associations between current or lifetime use of cannabis and IGT performance (Gonzalez et al., 2012; Verdejo-Garcia et al., 2013; Pacheco-Colón et al., 2019). For delay discounting, Strickland et al. reported a positive correlation between discounting rate and weekly use (Strickland et al., 2017), while Heinz et al. found no correlation with use (Heinz et al., 2013). Since cross-sectional data cannot resolve questions of causality, genetically informed studies coupled with longitudinal assessments will likely be required to determine whether cannabis use has a causal effect on decision making performance or impulsivity. Twin and familial studies suggest that genetic factors and shared environmental factors make major contributions to variations in cannabis use (Bogdan et al., 2016), delay discounting (Mitchell, 2019), IGT (Tuvblad et al., 2013) and trait impulsivity (Bezdjian et al., 2011). Longitudinal studies of twin pairs discordant for cannabis use have found little support for a causal association between cannabis use and impairments in general intelligence or executive function (Ross et al., 2020). To our knowledge, the decision making measures used in the present study have not been evaluated in a genetically informed design in cannabis users. However, an intriguing study of monozygotic twins discordant for cannabis use showed that cannabis using twins scored higher on self-report measures of experience seeking, boredom susceptibility and overall sensation seeking compared to non-using twins (Vink et al., 2007). Since monozygotic twins share 100% of genes and most aspects of familial environment, these results suggest that there may be a causal association between these individual personality characteristics and cannabis use, although the direction of influence remains to be characterized.

The role of altered decision making in substance use disorders has spurred development of interventions which target these processes. For example, Bickel et al. (2014) reported that working memory training reduced discounting rates in stimulant addicts. Similarly, Zhu et al. (2018) found that a mobile app designed to improve working memory and impulse control reduced delay discounting rates and improved IGT performance in persons with methamphetamine use disorder. Pharmacological interventions have rarely targeted decision making in substance use disorders, but animal studies and some pilot clinical trials provide support for this strategy (Perkins and Freeman, 2018; Chamberlain and Grant, 2019). The present results and previous findings in the literature should encourage testing of these novel therapies in cannabis use disorders as well.

The results of the present study should be interpreted in light of several limitations. First, the present cannabis user sample excluded subjects with comorbid psychiatric disorders. While this was intended to exclude individuals with other significant substance use or psychiatric histories that could obscure the potential effects of chronic cannabis use on task performance, it does limit the generalizability of the present findings. Second, the young age and high education level of this sample may have been protective factors which mitigated the effects of chronic cannabis exposure on the outcome measures. Third, the study was only powered to detect moderate to large effect sizes. A larger sample size might detect weak relationships between cannabis use and decision making measures. Fourth, quantity and frequency measures for the cannabis group were based upon participant report.

In summary, the results of this study indicate that chronic cannabis users showed poorer performance on two laboratory tests of decision-making (i.e., the IGT and Delay Discounting Task), as well as increased trait impulsivity. Cannabis users in this study did not show deficits on a measure of reversal learning and decision making under uncertainty (the PRLT), suggesting that heavy users' poorer decision making may not generalize to all measures of that construct. Targeting decision making in intervention trials could enhance treatment efficacy for cannabis use disorder.

## Data Availability Statement

The datasets presented in this study can be found in online repositories. The names of the repository/repositories and accession number(s) can be found at: https://doi.org/10.17605/OSF.IO/YGVQ7.

## Ethics Statement

The studies involving human participants were reviewed and approved by the Indiana University Human Subjects Committee and written informed consent was obtained from all participants. The patients/participants provided their written informed consent to participate in this study.

## Author Contributions

BO'D, PS, and DF were responsible for the design, execution, data analysis, interpretation, and manuscript preparation for this study. WH contributed to the design, interpretation, and manuscript preparation. All authors contributed to the article and approved the submitted version.

## Conflict of Interest

The authors declare that the research was conducted in the absence of any commercial or financial relationships that could be construed as a potential conflict of interest.
