# Supplementary material for: Decision Making and Impulsivity in Young Adult Cannabis Users
Source: Front Psychol. 2021 Jul 1;12:679904. doi: 10.3389/fpsyg.2021.679904 (PMC8280309; doi:10.3389/fpsyg.2021.679904)
Supplement: Supplementary file 1 [file Data_Sheet_1.docx]

**Supplementary Material**

Behavioral Task Methods

*Iowa Gambling Task (IGT; (Bechara et al 1994))*. For the IGT, participants were instructed that they would play a computerized card game in which their goal was to win as much money as possible. They were given a $2000 loan of play money at the start of the task and informed that they would earn a real-money bonus dependent upon how well they performed on the task. Participants were instructed that, on each trial, they were to choose a card from any one of the four decks (A, B, C, and D) shown face down on the screen using the mouse, that the computer would tell them the outcome of each choice, and that some decks were better than others. Each selection was associated with either a win of $100 (Decks A or B) or $50 (Decks C or D). Furthermore, 50% of selections from Deck A and Deck C were paired with small, frequent losses, whereas 10% of selections from Deck B and Deck D were paired with larger, less frequent losses. Selecting from Decks C and D (the advantageous decks) resulted in a net gain of $250 across 10 selections, whereas selecting from Decks A and B (the disadvantageous decks) resulted in a net loss of $250 across 10 selections. Participants were permitted to ponder their choices for as long as they wished before making a selection. Feedback regarding the amount won or lost was displayed for 2000 ms following each selection, and a cumulative win/loss tally for the session was displayed at the top of the screen and updated after each trial. The intertrial interval was 1000 ms. The placement of the decks on the screen and the sequence of cards in each deck was random across participants, but the decks were always labeled A-D from left to right on the screen. Participants who finished the task with a positive amount of money earned a real-money bonus for their performance, calculated in dollars as their final score divided by 500.

Participants completed 150 selections during the IGT, which was divided into 3 blocks of 50 trials for data analysis. For each participant, a “net score” (defined as the number of selections from advantageous decks minus the number of selections from disadvantageous decks) was calculated as an index of IGT performance for each block of 50 trials.

*Delay Discounting Task (DDT).* Participants completed a computerized version of the Delay Discounting Task (DDT). Prior to the start of the task, they were informed that they would be asked to make some hypothetical choices about money. On each trial, participants were presented with a choice between an amount of money available immediately or a larger amount of money available after some delay. Participants indicated their preferred (hypothetical) option by clicking the mouse on the appropriate button on the screen. The task consisted of 6 delay conditions presented in random order, and participants made 6 choices per condition. The delayed amount did change within each condition (e.g., “$800 available after 1 year”) but the immediately available amount was adjusted depending on the participants’ response on a given trial. The amount of the delayed reward was $800 for each condition with the amount of the immediately-available reward starting at $400 and the length of the delay period (2 weeks, 4 weeks, 6 months, 1 year, 3 years, or 10 years) varying across conditions. On each trial, the amount of the immediate reward changed depending on the participant’s response on the previous trial using an adjusting amount procedure (Green et al 2005); i.e., choosing the immediate reward on Trial *n* resulted in a decrease of the immediate reward amount offered on Trial *n* + 1, while choosing the delayed reward on Trial *n* resulted in an increase in the amount of the immediate reward offered on trial *n* + 1. The adjusting amount procedure was used to converge on an estimate of an immediately-available amount of money that the subject considered equivalent to the delayed amount.

The data from each participant was fit to the following hyperbolic delay discounting function (Green & Myerson 2004, Mazur 1987):

1. *V* = *A*/(1 + *kD*),

where *A* is the amount of the delayed reward (in the present study, $800), *D* is the delay until its receipt, *V* is subjective equivalent value of an immediate reward, and *k* is a free parameter corresponding to the individual’s discounting rate. Higher values of *k* correspond to steeper discounting of delayed rewards and a greater preference for immediate rewards. Each participant’s data was fit to Equation 1 using maximum likelihood estimation (Myung 2003) to estimate the free parameter, *k*. Discounting rates were positively skewed and were transformed using the natural logarithm prior to analysis (Kirby et al 1999).

*Probabilistic Reversal Learning task (PRLT)***.** For the PRLT, participants were instructed that their goal was to select one of two stimuli (different colored squares) on each trial and to determine, based upon feedback, which stimulus was usually correct. Participants also further instructed that once they determined which stimulus was usually correct they should choose that stimulus even if it was occasionally wrong, but that at some point the relationship between the stimuli in a pair might change so that the usually-correct stimulus became usually-incorrect and vice-versa. Participants were informed that each choice would be associated with a gain (if correct) or loss (if incorrect) of points, and that their goal was to win as many points as possible during the task. The PRLT consisted of three pairs of colored-square stimuli, with each pair corresponding to a single feedback condition. The feedback conditions were 100:0, 80:20, or 70:30, indicating the ratio of positive:negative (i.e., accurate:misleading) feedback provided upon selection of the correct stimulus from that pair). The stimuli that composed each pair were always presented together, with one stimulus presented on the right side of the computer monitor and the other on the left (determined randomly). Participants were instructed to indicate their selection by pressing a key corresponding to either the left or right stimulus. There was no time limit on selections, although participants were encouraged to respond as quickly and accurately as possible. The probabilistic nature of the feedback required participants to integrate knowledge of choice outcomes over several consecutive trials to correctly discriminate between the stimuli within each pair. Correct choices were followed by a message reading “CORRECT” and a gain of 100 points (positive feedback), whereas incorrect choices were followed by a message reading “WRONG” and a loss of 100 points (negative feedback). Feedback was presented for 1200 ms.

Each of the three stimulus pairs (feedback conditions) were presented for 80 trials. Participants responded to all 80 presentations of a given stimulus pair before a different pair was presented, and the order in which the pairs were presented was determined randomly prior to the start of the task. The criterion for demonstrating successful learning of each contingency condition was 8 consecutive correct responses to that pair. After that criterion was met the probability was 0.25 that the pair would reverse on the subsequent presentation. A pair did not reverse unless the participant had responded correctly during the last presentation of that pair. Following reversal, 8 consecutive correct responses to the pair were required before it reversed again. Each pair continued to reverse according to the criteria described above until all 80 presentations of that pair had been completed.

**Table S1.**  *Correlation coefficients between cannabis use measures and IGT, discounting rate and impulsivity*

___________________________________________________________________________

Duration of Use (Years) Frequency of Use (Past Month)

___________________________________________________

IGT Net Score -.13 -.23

Discounting Rate .06 .09

Barratt Impulsivity Score .20 .26

*Note*: Duration of Use and Frequency of Use refer to cannabis use. IGT: Iowa Gambling Task. None of the correlation coefficients were significant at *p* < .01.

**Cannabis Use Assessment Items**

**CANNABIS (MARIJUANA/HASHISH)**
*Note: For each question regarding cannabis use below, consider one instance of use as one session of attempting to get "high" or "intoxicated."*

Have you ever used cannabis (if no, please move on to the next item)?
 1 □ yes 2 □ no

During the last 6 months, have you used cannabis?
 1 □ yes 2 □ no

If yes, estimate how many times you have used cannabis (over the past 6 months)?

________________________­­­­­­­­­­­­­­­­­­­­_______________________________

During the last 3 months, have you used cannabis?
 1 □ yes 2 □ no

If yes, estimate how many times you have used cannabis (over the past 3 months)?

________________________­­­­­­­­­­­­­­­­­­­­_______________________________

During the last 30 days, have you used cannabis?
 1 □ yes 2 □ no

If yes, estimate how many times you have used cannabis (over the past 30 days)?

________________________­­­­­­­­­­­­­­­­­­­­_______________________________

During the past month, on average, how many **days per week** have you used cannabis?

________________________­­­­­­­­­­­­­­­­­­­­_______________________________

During the past month, on average, how many joints/bowls **per week** have you consumed?

(*note:* *1 joint = 1 bowl*)

________________________­­­­­­­­­­­­­­­­­­­­_______________________________

How many times have you used cannabis in the past week?

________________________­­­­­­­­­­­­­­­­­­­­_______________________________

On how many days have you had used cannabis in the past week?

________________________­­­­­­­­­­­­­­­­­­­­_______________________________

During the past week, how many joints/bowls have you consumed?

(*note:* *1 joint = 1 bowl*)

________________________­­­­­­­­­­­­­­­­­­­­_______________________________

How many days and hours has it been since you've last used cannabis?

*Days:_____________________ Hours:__­­­­­­­­­­­­­­­­­­­­_____________________*

At what age did you use cannabis for the first time?

 __________

Please estimate the time period in which you were using cannabis the most often:

*Years ago?_________________ Months ago? _________________*

During this period of heaviest use, on average, how much cannabis were you using?

*You got high _____ times per day?*

*You got high _____ times per week?*

*You got high _____ times per month?*

Approximately when did this period of heaviest use **begin**?

*Years ago?_________________ Months ago? _________________*

How long did this period of heaviest use **last**?

*How many years?______ or How many months?______ or How many days?______*

Approximately when did this period of heaviest use **end**?

*Years ago?_________________ Months ago? _________________*

Since this period of heaviest use ended, how much cannabis use have you averaged?

*You got high _____ times per day?*

*You got high _____ times per week?*

*You got high _____ times per month?*

To the best of your ability, please estimate how many times you've used cannabis in your lifetime?

________________________­­­­­­­­­­­­­­­­­­­­_______________________________

References

Bechara A, Damasio AR, Damasio H, Anderson SW. 1994. Insensitivity to future consequences following damage to human prefrontal cortex. *Cognition* 50: 7-15

Green L, Myerson J. 2004. A discounting framework for choice with delayed and probabilistic rewards. *Psychological Bulletin* 130: 769-92

Green L, Myerson J, Macaux EW. 2005. Temporal discounting when the choice is between two delayed rewards. *Journal of Experimental Psychology: Learning, Memory, and Cognition* 31: 1121-33

Kirby KN, Petry NM, Bickel WK. 1999. Heroin addicts have higher discount rates for delayed rewards than non-drug-using controls. *Journal of Experimental Psychology: General* 128: 78-87

Mazur JE. 1987. An adjusting procedure for studying delayed reinforcement In *Quantitative analyses of behavior: The effect of delay and of intervening events on reinforcement value*, ed. ML Commons, JE Mazur, JA Nevin, H Rachlin, pp. 55-73. Hillsdale, NJ: Erlbaum

Myung I. 2003. Tutorial on maximum likelihood estimation. *Journal of Mathematical Psychology* 47: 90-100
